# Protein Formulations Containing Polysorbates: Are Metal Chelators Needed at All?

**DOI:** 10.3390/antiox9050441

**Published:** 2020-05-20

**Authors:** Ema Valentina Brovč, Stane Pajk, Roman Šink, Janez Mravljak

**Affiliations:** 1 Faculty of Pharmacy, University of Ljubljana, Aškerčeva 7, SI-1000 Ljubljana, Slovenia; ema.valentina.brovc@ffa.uni-lj.si (E.V.B.); stane.pajk@ffa.uni-lj.si (S.P.); 2 Global Drug Development Technical Research and Development, Novartis, Biologics Technical Development Mengeš, Drug Product Development, Lek Pharmaceuticals d.d., Kolodvorska 27, SI-1234 Mengeš, Slovenia; roman.sink@novartis.com

**Keywords:** proteins, oxidation, Fenton reaction, free radicals, chelating agents, polysorbates

## Abstract

Proteins are prone to post-translational modifications at specific sites, which can affect their physicochemical properties, and consequently also their safety and efficacy. Sources of post-translational modifications include oxygen and reactive oxygen species. Additionally, catalytic amounts of Fe(II) or Cu(I) can promote increased activities of reactive oxygen species, and thus catalyse the production of particularly reactive hydroxyl radicals. When oxidative post-translational modifications are detected in the biopharmaceutical industry, it is common practice to add chelators to the formulation. However, the resultant complexes with metals can be even more damaging. Indeed, this is supported here using an ascorbate redox system assay and peptide mapping. Ethylenediaminetetraacetic acid (EDTA) addition strongly accelerated the formation of hydroxyl radicals in an iron-ascorbate system, while diethylenetriaminepentaacetic acid (DTPA) addition did not. When Fe(III) was substituted with Cu(II), EDTA addition almost stopped hydroxyl radical production, whereas DTPA addition showed continued production, but at a reduced rate. Further, EDTA accelerated metal-catalysed oxidation of proteins, and thus did not protect them from Fe-mediated oxidative damage. As every formulation is unique, justification for EDTA or DTPA addition should be based on experimental data and not common practice.

## 1. Introduction

Oxidation is one of the major chemical degradation pathways for protein pharmaceuticals, as it can result in covalent modifications to the amino-acid residues in proteins. Several amino acids are susceptible to oxidation. This includes, in particular, methionine and cysteine, and also histidine, tryptophan and tyrosine, due to their high reactivities with various reactive oxygen species. Oxidation of proteins can occur during the production steps, with oxygen concentrations that are too high or too low in cell culture media implicated in increased oxidative modifications [1,2,3]. As well as the production and purification processes, any excipients that are added during the formulation of a pharmaceutical and its subsequent storage conditions can directly or indirectly result in oxidative damage. For instance, polysorbates and polyethylene glycols can be contaminated with hydrogen peroxide (H_2_O_2_), and additionally, these excipients can undergo spontaneous autooxidation in the presence of dissolved oxygen, which can be accelerated by exposure to light, and which can produce hydroperoxides [4,5,6]. Although these are relatively stable species, trace amounts of transitional metals such as iron or copper (e.g., from stainless steel or metal affinity chromatography) can catalyse the conversion of these species into potent oxidants (e.g., hydroxyl radicals) [7,8,9,10]. Indeed, there are numerous studies on the metal-catalysed oxidation of proteins in the literature [11,12,13].

Zhou et al. studied the two metal chelators disodium ethylenediaminetetraacetic acid (Na_2_EDTA) and diethylenetriaminepentaacetic acid (DTPA) to define their stabilisation of a monoclonal antibody in solution formulations spiked with Fe(II). When the ratio of chelator to iron ions was >1, both Na_2_EDTA and DTPA reduced protein degradation. Under conditions of metal-catalysed oxidation, the incorporation of such chelating agents might reduce the rate of oxidation of proteins by scavenging the free metal ions before they oxidise the proteins [14]. Additionally, mannitol and EDTA are effective against H_2_O_2_- and Fe(II)-induced oxidation of proteins in solution [15].

However, it is not valid to assume that the addition of a chelating agent will eliminate oxidation, particularly if iron is the catalytic species. Kachur et al. reported that metal chelators such as EDTA and DTPA accelerated production of hydroxyl radicals (^•^OH) during autooxidation of ferrous ion (Fe(III)) complexes. If the Fe(III) complex is reduced to Fe(II) when oxygen is present, this results in the production of ^•^OH. This enhancement of autooxidation after chelation of iron was explained by alteration of the reaction mechanism from two-electron to one-electron reduction of oxygen molecules. The maximal yield of ^•^OH was seen with equal concentrations of chelator and Fe(II) [16,17].

Kocha et al. studied H_2_O_2_-mediated degradation of albumin. After exposure of albumin to the Fenton reaction between H_2_O_2_ and Fe(II)/(III), little ^•^OH was produced, and this was markedly increased by addition of EDTA [18]. Similarly, Fransson showed that oxidation of methionine only occurred with Fe(III) when EDTA was also added [19].

**Figure 1:**
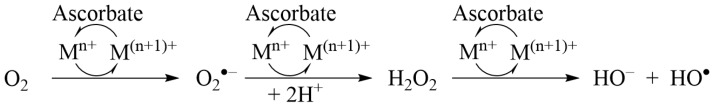
Metal-catalysed generation of reactive oxygen species (M^n+^: Cu(I) or Fe(II)).

The presence of oxygen is a necessary condition for protein oxidation. This oxygen can be reduced to the superoxide radical, and subsequently to H_2_O_2_, which is relatively stable (Figure 1). H_2_O_2_ can then be converted by the catalytic action of Cu(I) or Fe(II) to ^•^OH (Figure 1), which can then oxidise, and thus damage, amino acids in proteins. The influence of iron or copper chelation on the production of ^•^OH from H_2_O_2_ (i.e., the Fenton reaction) can be studied using an ascorbate redox system assay. In this assay, the ascorbate promotes reduction of Fe(III) to Fe(II), to maintain the Fe(II) levels. The Fe(II) can initially reduce oxygen to the superoxide radical. Subsequently, the Fe(II) reduces this to H_2_O_2_, and then finally converts H_2_O_2_ to the hydroxyl radical via the Fenton reaction (Figure 1) [20,21,22].

Unlike this ascorbate test system, the iron or copper in biopharmaceutical formulations can be reduced by, e.g., H_2_O_2_ or hydroperoxides that are introduced with the excipients [4,18]. Therefore, formulators tend to add chelators as a precautionary measure when oxidation of proteins is detected, such as EDTA or DTPA. However, previous studies on the influence of such chelating agents on the stability of pharmaceutical formulations have not been consistent as to whether EDTA and DTPA enhance or inhibit the Fenton reaction, and thus further studies are needed to clarify this.

Deleterious effects of reactive oxygen species do not end with protein and excipient oxidation. These oxidation products can react with the protein and impact upon its stability, and any resulting adducts can induce unwanted immune responses [23,24]. This is particularly relevant to the oxidation of polysorbates. These represent one of the most commonly used excipients in such biopharmaceutical formulations, which can result in the production of an array of potentially damaging products [25,26,27,28,29]. Ha et al. reported that polysorbate 80 (PS80) can increase oxidation products in an interleukin-2 formulation. This higher level of peroxides produced resulted in significant increases in oxidation in both the liquid and solid states. They also demonstrated that peroxides can be generated in pure PS80 in the presence of air at elevated temperatures [4]. Similarly, Knepp et al. demonstrated that alkyl hydroperoxides in PS80 can induce oxidation, dimerisation and subsequent aggregation of recombinant human ciliary neurotrophic factor [30].

Unlike oxidation of proteins, which is generally a one-off event in the absence of further production of reactive oxygen species, the oxidation of unsaturated fatty-acid esters of polysorbates can trigger lipid peroxidation, which is a cyclic process that can self-propagate. In particular, PS80 that contains higher levels of polyunsaturated fatty-acid esters is more prone to lipid peroxidation [31,32,33]. Many different chemical species can be produced as a result of polysorbates oxidation, including aldehydes, ketones and peroxides [27,28]. Detection of the production of such oxidation products is often problematic without their chemical derivatisation. Low molecular weight aldehydes and ketones show low ionisation efficiencies in mass spectrometry (MS) analysis due to their low proton affinities. Therefore, their chemical derivatisation is usually carried out with hydrazides, hydrazines, hydroxylamines or coumarines [29,34,35].

In the present study, we investigated the influence of metal ions, chelating agents and antioxidants on the rate of the Fenton reaction in a formulation of a therapeutic protein using the ascorbate redox system assay. Damage to the protein by reactive oxygen species was investigated using peptide mapping. Furthermore, the products of oxidative degradation of the polysorbate were derivatised and analysed using ultrahigh performance liquid chromatography with high-resolution MS (UHPLC-HRMS).

## 2. Materials and Methods

### 2.1. Solvents and Reagents

DTPA, EDTA, glutathione (GSH), quercetin, coumarine-3-carboxylic acid (CCA), 7-(diethylamino)coumarin-3-carbohydrazide (CHH), desferrioxamine, ascorbate, potassium phosphate (KH_2_PO_4_), and multi-compendial grade polysorbate 20 (PS20) and PS80 were from Sigma-Aldrich (Merck KgaA, Darmstadt, Germany). Copper(II) chloride and iron(III) chloride were from Acros Organics (Thermo Fischer Scientifics, Waltham, MA, USA). Ultra-gradient HPLC grade acetonitrile was from J.T. Baker (Avantor Performance Materials, Radnor, PA, USA). Liquid chromatography-MS grade methanol was from Merck KgaA (Darmstadt, Germany). Ultrapure water was from a Millipore Milli-Q water system (Merck KgaA, Darmstadt, Germany). The therapeutic protein solution was provided by Lek d.d. (Mengeš, Slovenia).

### 2.2. Ascorbate Redox System Assay

The promotion of ^•^OH production by chelating agents and antioxidants was evaluated in an ascorbate redox system assay, as described previously [20,36,37,38,39]. The Fe(III) chloride and Cu(II) chloride were dissolved and diluted in ultrapure water. All of the other solutions were prepared in 20 mM KH_2_PO_4_ buffer, pH 7.4. Each experiment was performed in triplicate using flat-bottomed 96-well black microtitre plates (Greiner Bio-One, Frickenhausen, Germany). For the hydroxyl radical, ^•^OH production was measured as the conversion of CCA into 7-hydroxy-CCA (λ_excitation_, 395 nm; λ_emission_, 450 nm). The general order of addition was as follows (in brackets are final concentrations): CCA (100 µM); chelating agent or antioxidant (EDTA, DTPA; GSH, quercetin) (10 µM); Cu(II) (10 µM) or Fe(III) (10 µM); and then following a 30 min incubation, ascorbate (300 µM). In selected samples, protein solution (250 µg/mL) or polysorbate solution (10 µg/mL) was added, and consequently the CCA volume was reduced (while maintaining the final incubation concentration). All of the test solutions also contained 1 µM desferrioxamine.

### 2.3. Peptide Mapping

Endoproteinase Lys-C was used as the proteolytic enzyme for digestion of the therapeutic protein. The resulting peptides were separated from the parent peptide using reverse-phase liquid chromatography. Quantification of relative amounts of oxidised methionine generated was through UV chromatography. The oxidative activities are expressed as the area under the chromatographic peak of the modified methionine produced relative to the sum of the areas under the total chromatographic peaks corresponding to the therapeutic peptide in each sample (i.e., modified plus unmodified). Chromatographic analysis was carried out using the method developed by Griffiths et al. [40].

The experimental additions to the therapeutic protein solution in these incubations thus included (in brackets are final concentrations): no further additions (control); Fe(III) (0.1 mM) without and with ascorbate (3 mM); Fe(III) (0.1 mM) and EDTA (0.1 mM) without and with ascorbate (3 mM). All of the stock solutions were prepared in Tris/HCl buffer, except for the Fe(III) (ultrapure water). All samples were incubated at 37 °C for 42 h.

### 2.4. Oxidation of Polysorbates

Polysorbates PS20 and PS80 (10 mmol/L) were incubated in a mixture of ethanol and water (10:90; *v/v*) containing 0.5 mmol/L copper(II) sulphate and 1 mmol/L ascorbate at 37 °C on an orbital shaker for 72 h. After this oxidation, the low and high molecular weight carbonylated compounds produced were derivatised with CHH and analysed immediately by direct injection into the high-resolution positive electrospray ionisation-MS, as described by Milic et al. [35]. Moreover, the compositions of the non-derivatised oxidised polysorbate samples were analysed using UHPLC-HRMS.

### 2.5. Polysorbate Composition Profile

Mass spectrometry (Q Exactive Plus Hybrid Quadrupole-Orbitrap mass spectrometer; Thermo Fischer Scientific Inc., Waltham, MA, USA) was performed under the following basic MS parameters: sheath gas flow rate, 25 (arbitrary units); auxiliary gas flow rate, 10 (arbitrary units); capillary temperature, 350 °C; and spray voltage, 3.5 kV. The MS was operated in positive electrospray ionisation. The compounds were analysed using a C18 reversed-phase column (Poroshell 300SB; 5 µm particle size; 2.1 mm × 75 mm; Agilent, Santa Clara, CA, USA) at a column temperature of 30 °C and a flow rate of 0.7 mL/min. The injection volume was 5 µL, with a total run time of 45 min. Chromatographic analysis was based on the modified method of Fekete et al. [41].

## 3. Results and Discussion

### 3.1. Ascorbate Redox System Assay

**Figure 2:**
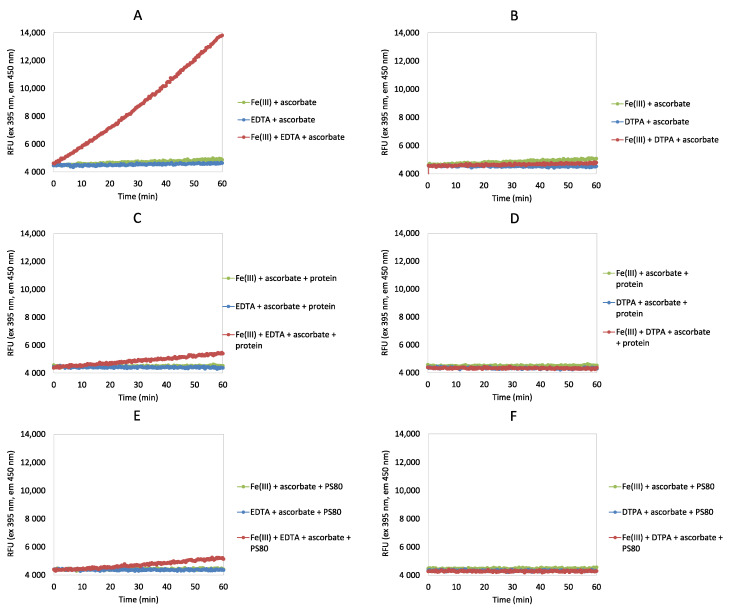
Time courses of the production of ^•^OH (RFU, relative fluorescence units) during autooxidation of Fe(III) complexes measured according to the fluorescence intensities of 7-hydroxy-coumarine-3-carboxylic acid after incubations of CCA with ascorbate ± Fe(III), and ± ethylenediaminetetraacetic acid (EDTA) (**A**,**C**,**E**) or ± diethylenetriaminepentaacetic acid (DTPA) (**B**,**D**,**F**), and plus protein (**C**,**D**) or plus PS80 (**E**,**F**). Positive control, ascorbate and Fe(III), and plus protein (**C**,**D**) or plus PS80 (**E,F**); negative control, ascorbate plus chelating agent (EDTA/DTPA) or antioxidant (quercetin/GSH), and plus protein (**C,D**) or plus PS80 (**E,F**); see also Figures S1, S3 and S5. All solutions were prepared in 20 mM KH_2_PO_4_, 1 µM desferrioxamine, pH 7.4, except FeCl_3_ (ultrapure water).

**Figure 3:**
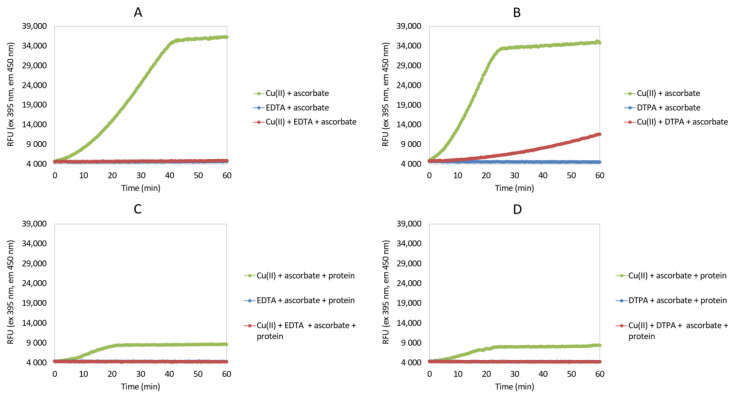
Time courses of the production of ^•^OH (RFU, relative fluorescence units) during autooxidation of Cu(II) complexes measured according to the fluorescence intensity of 7-hydroxy-coumarine-3-carboxylic acid after incubation of CCA with ascorbate ± Cu(II), and ± EDTA (**A**,**C**) or ± DTPA (**B**,**D**), and plus protein (**C**,**D**). Positive control, ascorbate plus Cu(II), and plus protein (**C**,**D**); negative control, ascorbate plus chelating agent (EDTA/DTPA) or antioxidant (quercetin/GSH), and plus protein (**C**,**D**); see also Figures S2 and S4. All solutions were prepared in 20 mM KH_2_PO_4_, 1 µM desferrioxamine, pH 7.4, except CuCl_2_ (ultrapure water).

First, the production of ^•^OH during autooxidation of Fe(III) and Cu(II) complexes was investigated in the ascorbate redox system assay in the absence and presence of different chelating agents (i.e., EDTA, DTPA) and antioxidants (i.e., GSH, quercetin) (Figure 2 and Figure 3; Figures S1 and S2). Furthermore, the addition of protein under the same Fe(III) and Cu(II) conditions was evaluated (Figure 2 and Figure 3; Figures S3 and S4), as well as the impact of PS80 addition under these Fe(III) conditions (Figure 2; Figure S5).

Production of ^•^OH in the presence of Fe(III) ions was strongly accelerated only by addition of EDTA, while addition of DTPA (Figure 2) or antioxidants (Figure S1) to the ascorbate assay showed similar responses to the negative controls. This appears to occur through iron cycling via iron complexation (Figure 1). In the presence of protein, the production of ^•^OH was less pronounced, which will be due to the competition of the protein with CCA for ^•^OH. In the absence of EDTA, the reaction was slower or inhibited fully. Similar to the presence of protein, PS80 addition also resulted in lower ^•^OH production, as PS80 is highly susceptible to radical attack, and will therefore compete with CCA for ^•^OH. PS80 contains saturated and unsaturated fatty acids, and its free-radical-initiated mechanism is well established, which consists of the chain initiation, propagation and termination steps. In order to compare the results more easily, initial reaction rates have been calculated (Table S1).

In contrast to the use of Fe(III) ions in the ascorbate assay, Cu(II) ions have greater reactivity and accelerated the reaction also in the absence of the chelators EDTA and DTPA (Figure 3). Interestingly, the addition of EDTA in combination with Cu(II) completely inhibited ^•^OH production. Addition of DTPA here showed some inhibition of ^•^OH production, although this remained greater compared to EDTA. Addition of the reducing reagents quercetin and GSH had no impacts on the Cu(II)-induced autooxidation here, with similar responses to the positive control (Figure S2). As already shown for Fe(III), the addition of protein lowered the production of ^•^OH in Cu(II), which was completely inhibited here by the addition of either EDTA or DTPA with the protein (Figure 3).

According to the data obtained here, as well as the concentration of the complex, the rate of generation of ^•^OH strongly depends on the metal ion and the chelator used. Comparing Fe(III) and Cu(II), the efficiency of EDTA and DTPA to accelerate the Fenton reaction for the production of ^•^OH was not equivalent. With Fe(III), EDTA strongly accelerated ^•^OH production, whereas DTPA slowed it with both Fe(III) and Cu(II). With the addition of protein or PS80, the production of ^•^OH has been less pronounced because the protein/PS80 will have competed with CCA for ^•^OH. The main advantage of ascorbate redox system assay is the simplicity of the test, rapid results and small amount of sample needed. However, the change in the rate of CCA production does not provide any solid information about the underlaying mechanism. For a more detailed interpretation of the ascorbate redox system assay, a method with higher specificity should be used (e.g., peptide mapping).

### 3.2. Peptide Mapping

Peptide mapping is a technique for the identification and characterisation of proteins and their modifications. Peptide mapping methods have been specifically developed to locate protein regions (i.e., amino acids) that have been altered (e.g., oxidised). It is well known that the susceptibility to oxidation of methionine residues depends on their location within the structure of a protein. Oxidation of methionine side chains can be mediated by double electron transfer (e.g., by non-radical oxidants, such as peroxides) or single electron transfer (e.g., by metal catalysis) [42], although here we focussed only on single electron transfer with iron.

Most chelators result in equimolar complexes with iron [16]. Zhou et al. showed that EDTA and DTPA cannot protect the degradation of a protein by iron if the ratio of the molar concentrations of the metal chelator to iron is less than 1:1 (i.e., an excess of chelator is required) [14]. Therefore, in the present study, equal concentrations of iron and chelator were used for the oxidation, although with a 30-fold excess of ascorbate over iron, to ensure that most of the iron was in the ferrous (Fe(II)) form.

**Figure 4:**
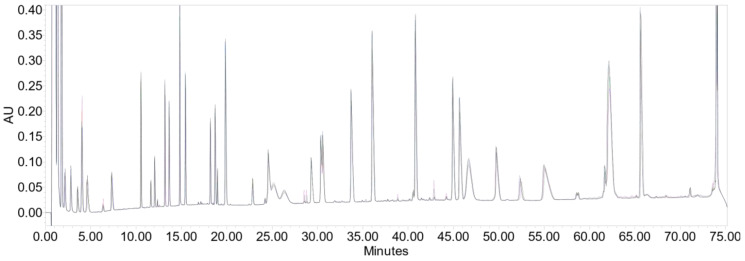
HPLC-UV peptide map of the therapeutic protein. Fragments were prepared by protein digestion, as described in the Materials and Methods Section.

Using peptide mapping, we resolved the oxidised peptides from those that were not oxidised (Figure 4). Moreover, we measured protein oxidation under the various stress conditions. The optimum choice of chemical additives needs to be evaluated on the basis of the specific protein oxidation mechanism. The different conditions investigated to induce oxidation of methionine in the protein solution were thus: without additives (control), Fe(III) without and with ascorbate, Fe(III) and EDTA without and with ascorbate.

**Figure 5:**
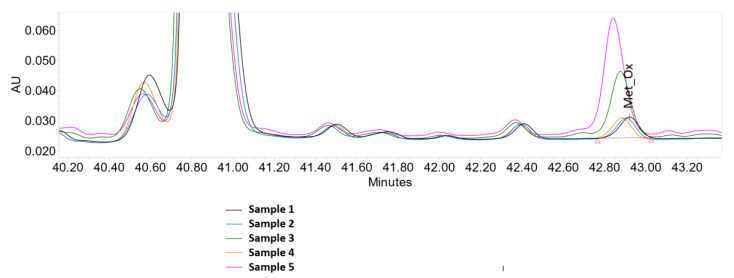
HPLC-UV peptide map of the degraded and non-degraded protein considering the oxidised methionine peaks indicated (Met_ox). The five experimental conditions (‘sample names’) are as indicated in Table 1.

These data showed that Fe(III) or EDTA alone did not trigger the oxidation, and that there is the need to include a reducing agent (e.g., ascorbate) and a chelating agent (e.g., EDTA) for high levels of oxidation (Figure 5; Table 1).

**Figure 6:**
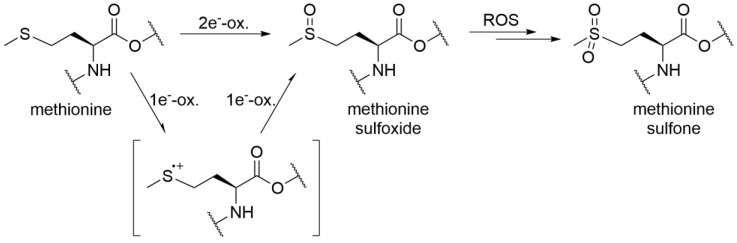
Pathways of methionine oxidation.

As reported previously, methionine oxidation (Figure 6) in protein biopharmaceuticals has been correlated with number of adverse effects, whereby the therapeutic protein can undergo a loss of function, loss of activity and/or increase in aggregation propensity. These processes, therefore, need to be carefully controlled [43,44]. During the protein processing and storage, the levels of transition metals should be monitored and the addition of chelating agents should be considered. Additionally, EDTA can facilitate migration of metal ions from metal contact surfaces into solution, as reported by Kocijan et al. [45] and Zhou et. al. [46]. This can thus be important for the stability of a therapeutic protein, as we have shown here that addition of EDTA can enhance its oxidative degradation. Therefore, the addition of chelating agents to the therapeutic protein formulation should be carefully considered and the final decision supported by experimental data.

### 3.3. Polysorbates Degradation: Oxidation of Polysorbates and Their Composition Profiles

The stabilising properties of polysorbates in solution can be overshadowed by their susceptibility to autooxidation. The major sites for initiation of autooxidation for polysorbates include unesterified polyoxyethylenes [25], ester bonds [47] and the unsaturation sites of fatty acids [32,48]. Polysorbates are mixtures of various fatty acid esters. The main fatty acid of the multi-compendial grade PS20 is lauric acid (C12:0), which represents 40% to 60% of the total number of free fatty acid species, whereas in PS80 it is predominantly oleic acid (C18:1) with ≥58%. Besides, high levels of monounsaturated oleic acid and also relatively high levels of long-chain polyunsaturated fatty acids are presented in multi-compendial PS80, such as linoleic acid (C18:2; ≤18%) and linolenic acid (C18:3; ≤4%) [49].

Due to the larger number of oxidisable sites in the fatty-acid chains of PS80 versus PS20, PS80-containing formulations are known to be more susceptible to oxidative degradation than PS20 formulations [4,27]. Moreover, lower levels of polyunsaturated fatty acids contribute less to oxidative degradation than higher levels monounsaturated oleic acid [50]. In contrast to PS80, the α-carbons of polyoxyethylenes are the most susceptible for oxidation in PS20 [25,48].

**Figure 7:**
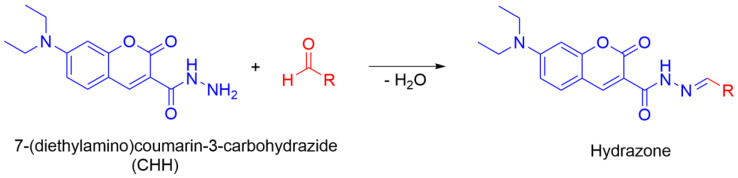
Derivatization reaction of oxidation products of polysorbate with CHH.

**Figure 8:**
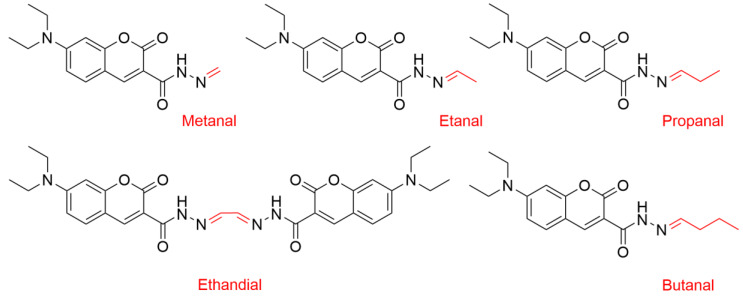
The most common oxidation products identified here from PS20 and PS80 after derivatisation with CHH. The additional oxidation products identified are shown in Figures S6 (PS20) and S7 (PS80).

We also studied here the influence of Cu(II) ions and ascorbate on the oxidation of the PS20 and PS80 polysorbates. After oxidation, the samples were derivatised with CHH for the MS analysis (Figure 7 and Figure 8). According to the data from the ascorbate redox assays, oxidation with Cu(II) was faster compared to that for Fe(III). Moreover, there was no need to accelerate the copper redox cycling by metal complexation. The method was developed according to the lipid peroxidation method of Milic et al. [35], who studied polyunsaturated fatty acids in lipids. This was thus successfully adopted to testing the stability of polysorbates here for the first time, and this might prove to be very useful in the future for monitoring oxidative degradation of polysorbates.

For PS20, 18 oxidation products were identified here (Figure 8; Figure S6), with more than twice (*n* = 39) identified from PS80 (Figure 8; Figure S7). Indeed, PS80 contains higher levels of long-chain fatty-acid esters compared to PS20, and it is therefore more prone to oxidation. As expected, the greater the number of double bounds in the polysorbates, the higher their susceptibility to oxidation.

Cleavage of the oleic acid double bond mainly produced low-molecular-weight aldehydes (≤C8), which are also more reactive than long-chain aldehydes. Additionally, during the polysorbates degradation, the oxidation products might themselves induce direct changes in the protein structure, which can result in less protein stabilisation.

**Figure 9:**
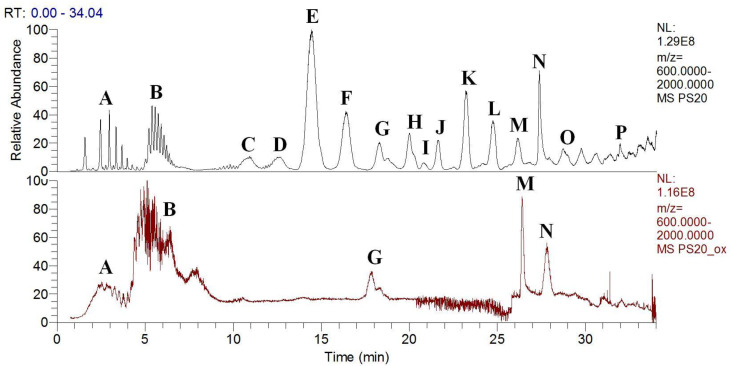
Total ion current (Mr ≥ 600 Da) chromatograms of non-degraded (top) and degraded (bottom) PS20 after oxidation with Cu(II) and ascorbate, and following UHPLC-HRMS. The subspecies in the non-degraded PS20 were identified as: **A**, unesterified polyoxyethylene isosorbides; **B**, unesterified polyoxyethylene sorbitans; **C**, monoesters with caprylic acid; **D**, monoesters with capric acid; **E**, monoesters with lauric acid; **F**, monoesters with myristic acid; **G**, monoesters with palmitic acid; **H**, monoesters with oleic acid; **I**, diesters with lauric and caprylic acid; **J**, diesters with lauric and capric acid; **K**, diesters with double lauric acid; **L**, diesters with lauric and myristic acid; **M**, diesters with lauric and palmitic acid; **N**, diesters with lauric and oleic acid; **O**, triesters of polyoxyethylene sorbitan; **P**, tetraesters of polyoxyethylene sorbitan.

With the higher content of different fatty acids in PS20 compared to PS80, the profile of the PS20 composition from the chromatograms was more complex. Using reverse-phase chromatography, the subspecies in the non-degraded and degraded PS20 were separated and eluted, as shown in Figure 9. After the oxidation of PS20 (Figure 9, bottom), all of the major peaks were absent. Monoesters with lauric acid were the most abundant in the non-degraded PS20 (Figure 9, top), whereas in the degraded PS20, aside from the most abundant non-esterified species, only low levels of mono- and diesters with lauric, palmitic and oleic acid remained (Figure 9, bottom).

**Figure 10:**
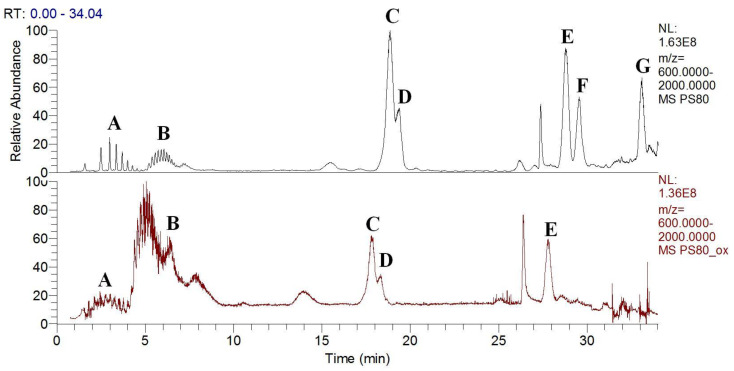
Total ion current (Mr ≥ 600 Da) chromatograms of non-degraded top) and degraded (bottom) PS80 after oxidation with Cu(II) and ascorbate, and following UHPLC-HRMS. The subspecies in the non-degraded PS80 were identified as: **A**, unesterified polyoxyethylene isosorbides, unesterified polyoxyethylenes; **B**, unesterified polyoxyethylene sorbitans; **C**, monoesters of polyoxyethylene sorbitan, polyoxyethylene monoesters; **D**, monoesters of polyoxyethylene isosorbide; **E**, diesters of polyoxyethylene sorbitan; **F**, diesters of polyoxyethylene isosorbide; **G**, triesters of polyoxyethylene sorbitan.

Again, using reverse-phase chromatography, the subspecies in the non-degraded and degraded PS80 were eluted and identified, as shown in Figure 10. For the non-degraded PS80, the monoesters were the most abundant, followed by the diesters and then the triesters (Figure 10, top). In contrast, in the degraded PS80, the non-esterified species were the most abundant, while the proportions of monoesters and polyesters were greatly lowered, and the triesters were completely degraded (Figure 10, bottom). The whole chromatogram was also slightly shifted for the degraded PS80.

## 4. Conclusions

To avoid stability problems for protein solutions containing polysorbates, the levels of transition metals should be carefully controlled during protein processing and storage, and the addition of chelating agents can be considered. According to the data from the present study, it is not valid to assume that the addition of chelators will eliminate protein oxidation, particularly if iron, rather than copper, is the contaminating metal. The oxidation of methionine was catalysed by dissolved oxygen, Fe(III) and ascorbate, and this was accelerated on addition of EDTA. As for protein solutions, metal ions in combination with ascorbate can also be problematic for polysorbate solutions. Any addition of chelating agents to the therapeutic protein formulations should be based on experimental data confirming the beneficial effect of such an addition. Using UHPLC-HRMS, we defined and compared the composition profiles of the non-degraded and degraded PS20 and PS80 polysorbates, with the various degradation products of this metal-catalysed oxidation identified. This method developed here for polysorbates derivatisation should be of great use in future stability studies for the monitoring of polysorbates oxidation.

## Figures and Tables

**Table 1:** Peptide mapping analysis for the methionine oxidation, as determined by HPLC-UV (e.g., Figure 5).

Sample	Composition	Met_Ox
1	control sample (without additives)	1.8
2	Fe(III)	1.7
3	Fe(III) + ascorbate	6.4
4	Fe(III) + EDTA	1.8
5	Fe(III) + EDTA + ascorbate	12.9

## Supplementary Materials

The following are available online at https://www.mdpi.com/2076-3921/9/5/441/s1.
