# Supplementary material for: Protein Formulations Containing Polysorbates: Are Metal Chelators Needed at All?
Source: Antioxidants (Basel). 2020 May 20;9(5):441. doi: 10.3390/antiox9050441 (PMC7278585; doi:10.3390/antiox9050441)
Supplement: Supplementary file 1 [file antioxidants-09-00441-s001.pdf]

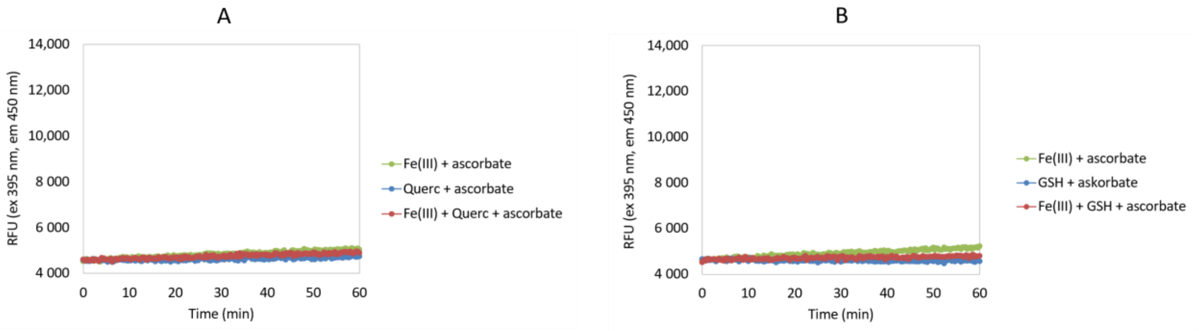

Figure S1. Time courses of the production of •OH (RFU, relative fluorescence units) during autooxidation of Fe(III) complexes measured according to the fluorescence intensities of 7-hydroxy-coumarine-3-carboxylic acid after incubations of CCA with ascorbate ± Fe(III), and ±quercetin (Querc) **(A)** and ± glutathione (GSH) **(B)**. The positive control included ascorbate and Fe(III), and the negative control included ascorbate and antioxidant/chelating agent (see also Figure 2 for EDTA/DTPA). All solutions were prepared in 20 mM KH_2PO_4, 1 µM desferrioxamine, pH 7.4, except $FeCl_3$ (ultrapure water).

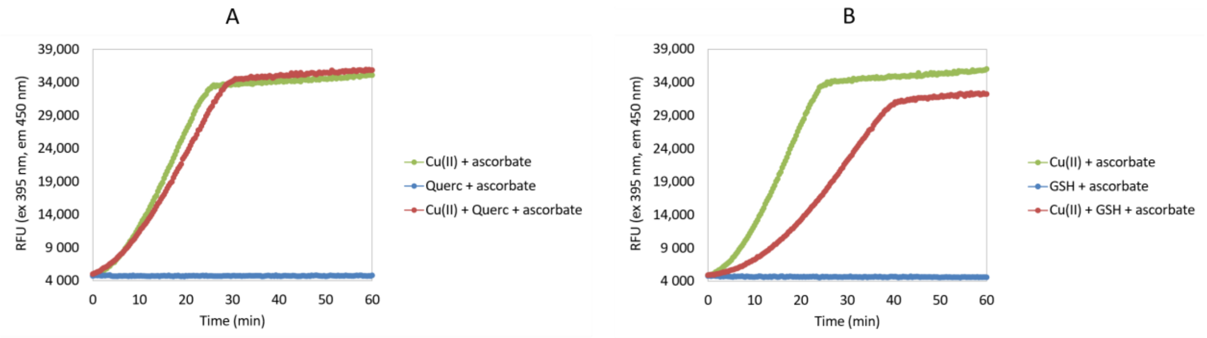

Figure S2. Time courses of the production of $^{\bullet}OH$ (RFU, relative fluorescence units) during autooxidation of Cu(II) complexes measured according to the fluorescence intensities of 7-hydroxy-coumarine-3-carboxylic acid after incubations of CCA with ascorbate ± Cu(II), and ± quercetin (Querc) **(A)** and ± glutathione (GSH) **(B)**. The positive control included ascorbate and Cu(II), and the negative control included ascorbate and antioxidant/chelating agent (see also Figure 3 for EDTA/DTPA). All solutions were prepared in 20 mM KH_2PO_4, 1 μM desferrioxamine, pH 7.4, except $CuCl_2$ (ultrapure water).

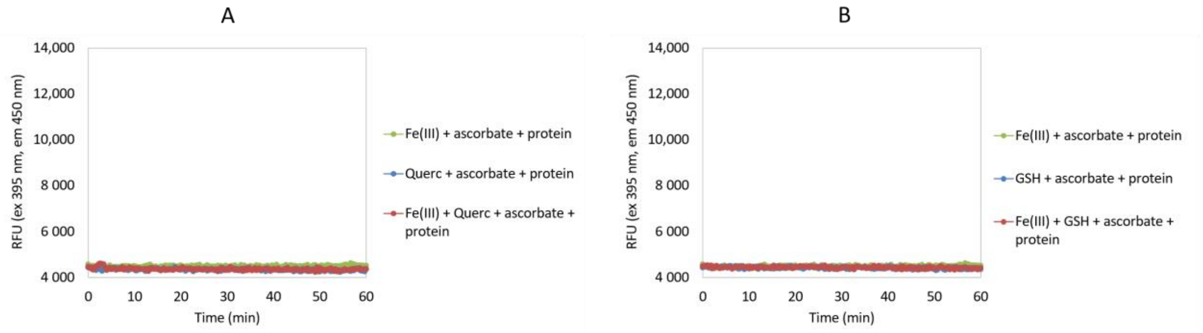

Figure S3. Time courses of the production of •OH (RFU, relative fluorescence units) during autooxidation of Fe(III) complexes measured according to the fluorescence intensities of 7-hydroxy-coumarine-3-carboxylic acid after incubations of CCA with ascorbate and protein ± Fe(III), and ± quercetin (Querc) **(A)** and ± glutathione (GSH) **(B)** (see also Figure 2 for EDTA/DTPA).

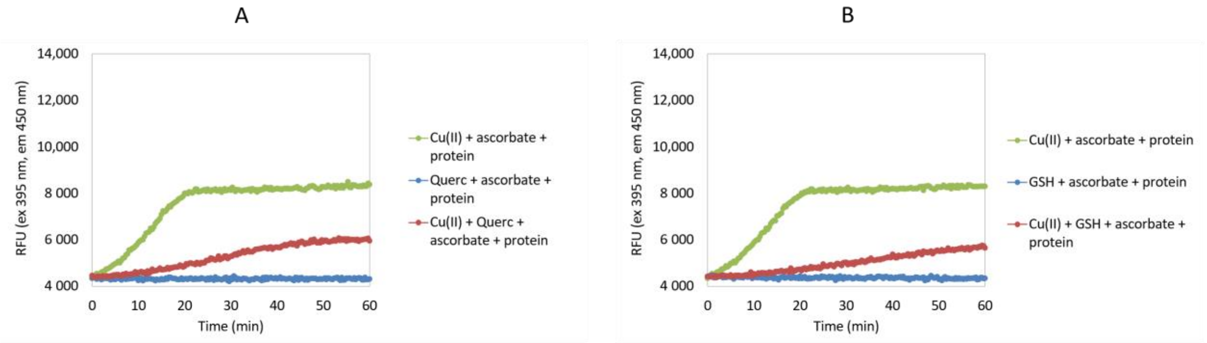

Figure S4. Time courses of the production of •OH (RFU, relative fluorescence units) during autooxidation of Cu(II) complexes measured according to the fluorescence intensities of 7-hydroxy-coumarine-3-carboxylic acid after incubations of CCA with ascorbate and protein ± Cu(II), and ± quercetin (Querc) **(A)** and ± glutathione (GSH) **(B)** (see also Figure 3 for EDTA/DTPA).

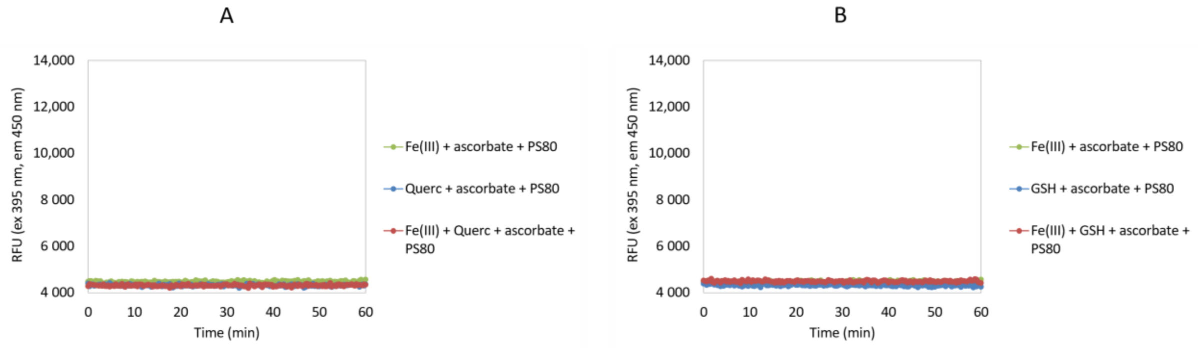

Figure S5. Time courses of the production of •OH (RFU, relative fluorescence units) during autooxidation of Fe(III) complexes measured according to the fluorescence intensities of 7-hydroxy-coumarine-3-carboxylic acid after incubations of CCA with ascorbate and PS80 ± Fe(III), and ±quercetin (Querc) **(A)** and ± glutathione (GSH) **(B)** (see also Figure 2 for EDTA/DTPA).

Table S1. Initial reaction rates of ascorbate redox system assays.

Sample composition	Initial reaction rate (RFU/min)
Fe(III) + ascorbate (positive control)	7.16
EDTA + ascorbate (negative control)	2.85
Fe(III) + EDTA + ascorbate	174
Fe(III) + ascorbate (positive control)	9.74
DTPA + ascorbate (negative control)	1.61
Fe(III) + DTPA + ascorbate	5.88
Fe(III) + ascorbate (positive control)	8.29
Querc + ascorbate (negative control)	2.80
Fe(III) + Querc + ascorbate	5.76
Fe(III) + ascorbate (positive control)	8.85
GSH + ascorbate (negative control)	-0.74
Fe(III) + GSH + ascorbate	2.68
Fe(III) + ascorbate + protein (positive control)	0.62
EDTA + ascorbate + protein (negative control)	-0.64
Fe(III) + EDTA + ascorbate + protein	16.67
Fe(III) + ascorbate + protein (positive control)	0.61
DTPA + ascorbate + protein (negative control)	-1.20
Fe(III) + DTPA + ascorbate + protein	-0.66
Fe(III) + ascorbate + protein (positive control)	0.63
Querc + ascorbate + protein (negative control)	-1.10
Fe(III) + Querc + ascorbate + protein	-1.42
Fe(III) + ascorbate + protein (positive control)	0.62
GSH + ascorbate + protein (negative control)	-0.96
Fe(III) + GSH + ascorbate + protein	-1.31
Fe(III) + ascorbate + PS80 (positive control)	-0.51
EDTA + ascorbate + PS80 (negative control)	-0.44
Fe(III) + EDTA + ascorbate + PS80	14.9
Fe(III) + ascorbate + PS80 (positive control)	0.50
DTPA + ascorbate + PS80 (negative control)	-0.51
Fe(III) + DTPA + ascorbate + PS80	-0.06
Fe(III) + ascorbate + PS80 (positive control)	0.50
Querc + ascorbate + PS80 (negative control)	-0.26
Fe(III) + Querc + ascorbate + PS80	0.20
Fe(III) + ascorbate + PS80 (positive control)	0.51
GSH + ascorbate + PS80 (negative control)	-0.14
Fe(III) + GSH + ascorbate + PS80	-0.66
Cu(II) + ascorbate (positive control)	973
EDTA + ascorbate (negative control)	1.47
Cu(II) + EDTA + ascorbate	4.14
Cu(II) + ascorbate (positive control)	1470
DTPA + ascorbate (negative control)	-1.79
Cu(II) + DTPA + ascorbate	162
Cu(II) + ascorbate (positive control)	1500
Querc + ascorbate (negative control)	0.14
Cu(II) + Querc + ascorbate	1240
Cu(II) + ascorbate (positive control)	1550
GSH + ascorbate (negative control)	-2.04
Cu(II) + GSH + ascorbate	912
Cu(II) + ascorbate + protein (positive control)	219
EDTA + ascorbate + protein (negative control)	-0.77
Cu(II) + EDTA + ascorbate + protein	-0.93
Cu(II) + ascorbate + protein (positive control)	218
DTPA + ascorbate + protein (negative control)	-0.75
Cu(II) + DTPA + ascorbate + protein	-0.92
Cu(II) + ascorbate + protein (positive control)	219
Querc + ascorbate + protein (negative control)	-0.07
Cu(II) + Querc + ascorbate + protein	39.6
Cu(II) + ascorbate + protein (positive control)	220
GSH + ascorbate + protein (negative control)	-1.12
Cu(II) + GSH + ascorbate + protein	26.7

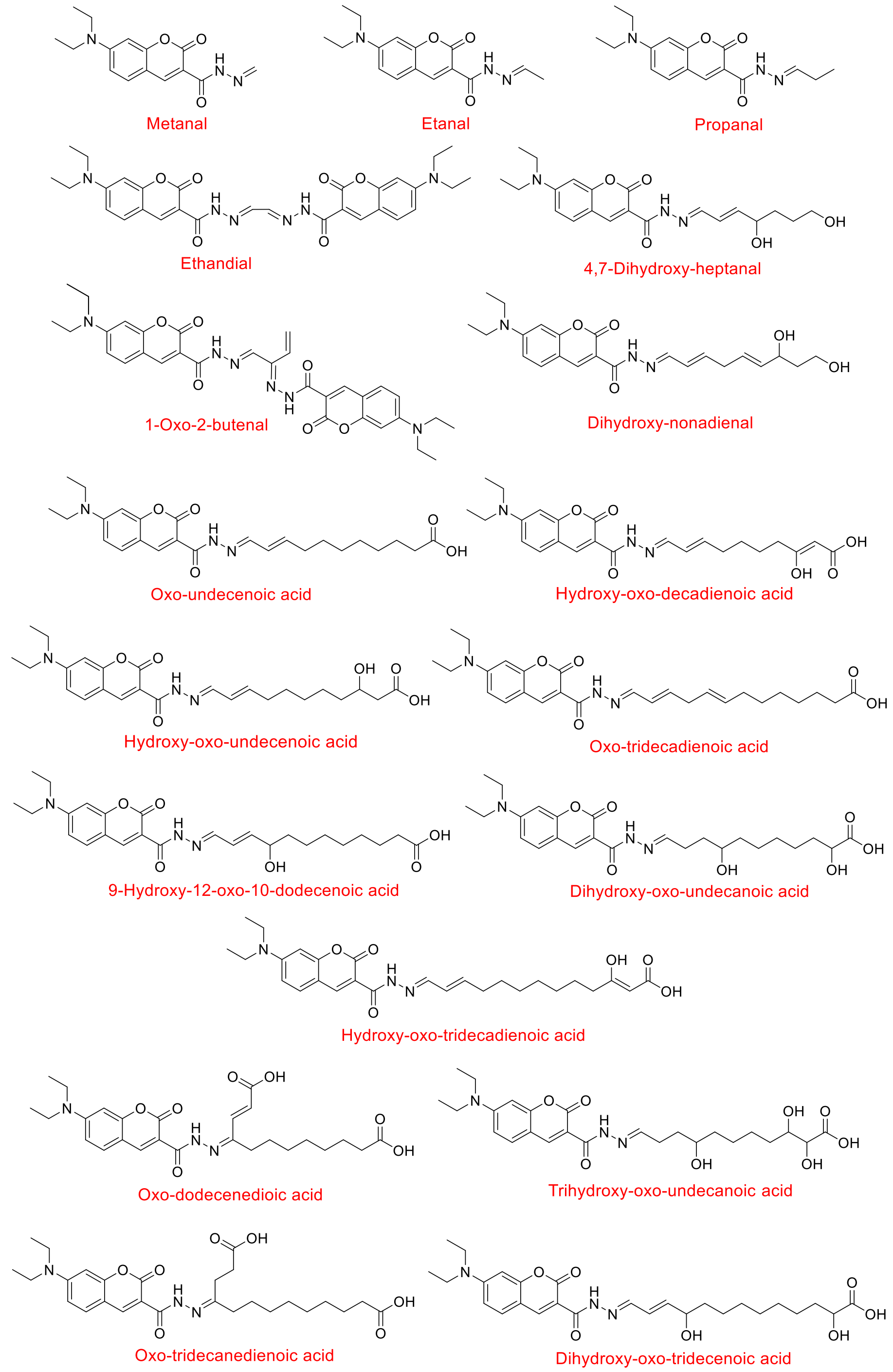

Figure S6. Oxidation products of PS20 identified using high-resolution mass spectrometry.

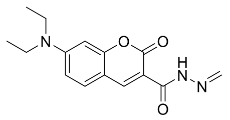

Metanal

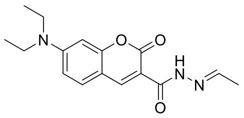

Etanal

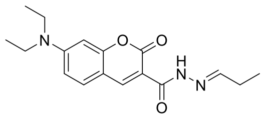

Propanal

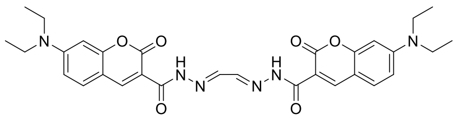

Ethandial

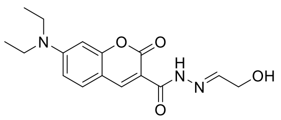

2-Hydroxy-ethanal

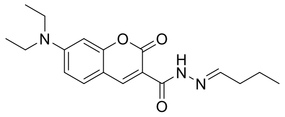

Butanal

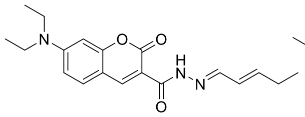

2-Pentenal

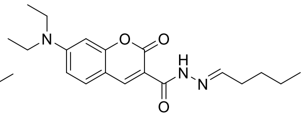

Pentanal

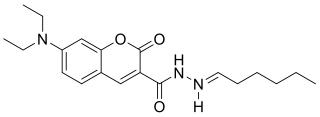

Hexanal

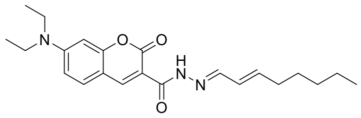

Octenal

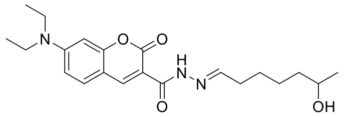

6-Hydroxy-heptanal

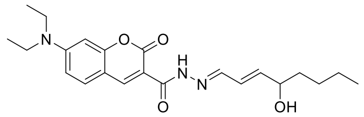

Hydroxy-octenal

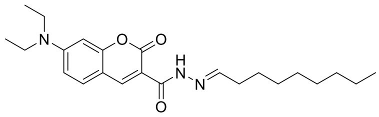

Nonanal

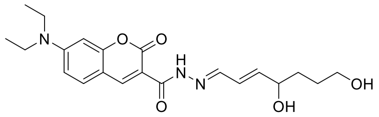

4,7-Dihydroxy-heptenal

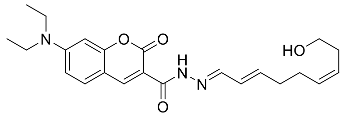

Hydroxy-2,6-nonadienal

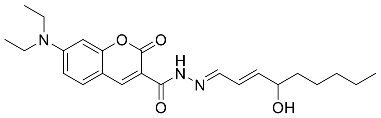

4-Hydroxy-2-nonenal

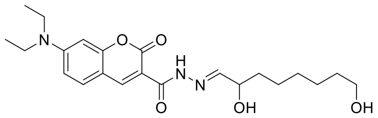

Dihydroxy-octanal

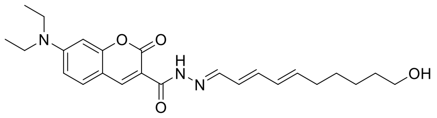

Hydroxy-decadienal

[continued]

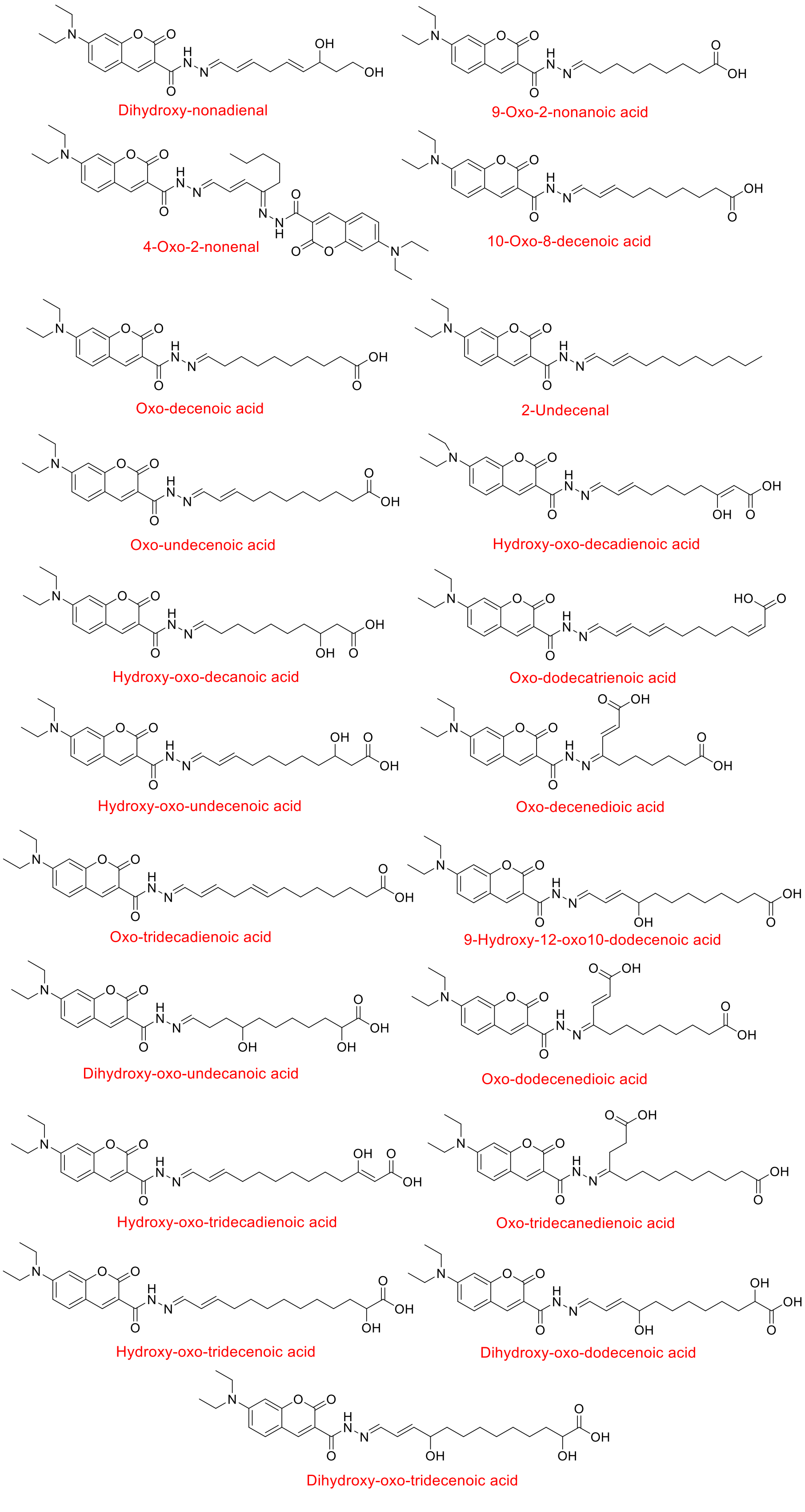

Figure S7. Oxidation products of PS80 identified using high-resolution mass spectrometry.
